# Empagliflozin Effect on Left Cardiac Parameters in Acute Coronary Syndrome: A Systematic Review and Meta-Analysis of Randomized Controlled Trials

**DOI:** 10.7759/cureus.69229

**Published:** 2024-09-11

**Authors:** Ghaith Y Eljadid, Mohamed Saad Rakab, Ahmed Mansour, Nereen A Almosilhy, Ahmed W Abbas, Noura Abdrabou, Amira S Alarab, Yasmeen Abdelglel Mohamed, Ahmed Khaled, Elarbi Goufa, Ahmed Elbataa, Heba A Aboeldahab

**Affiliations:** 1 Faculty of Medicine, Ain Shams University, Cairo, EGY; 2 Faculty of Medicine, Mansoura University, Mansoura, EGY; 3 Faculty of Medicine, Al-Azhar University, Cairo, EGY; 4 Faculty of Pharmacy, Tanta University, Tanta, EGY; 5 Faculty of Medicine, Alexandria University, Alexandria, EGY; 6 Faculty of Medicine, University of Aleppo, Aleppo, SYR; 7 Faculty of Medicine, Cairo University, Cairo, EGY; 8 Faculty of Medicine, University of Beni Suef, Beni Suef, EGY; 9 Faculty of Medicine, University of Oran 1 - Ahmed Ben Bella, Oran, DZA; 10 Faculty of Pharmacy, Alexandria University, Alexandria, EGY

**Keywords:** acute coronary syndrome, cardiac parameters, empagliflozin, meta-analysis, myocardial infarction, sglt2, ventricular remodeling

## Abstract

Acute coronary syndrome (ACS) poses a significant global health burden despite advancements in its management. Sodium-glucose cotransporter 2 (SGLT2) inhibitors, primarily used in type 2 diabetes mellitus (T2DM), have gained recent consideration as potential agents for ACS management due to their cardiovascular benefits beyond glycemic control. This study aimed to assess the effects of empagliflozin on left cardiac parameters in ACS patients. PubMed, Cochrane, Scopus, and Web of Science were searched thoroughly to identify relevant randomized controlled trials (RCTs). Four RCTs involving 701 patients were included. Compared to placebo, empagliflozin significantly reduced left ventricular end-systolic volume index (mean difference (MD): -2.38, 95% CI: -3.95 to -0.80, p = 0.0032), left ventricular mass index (MD: -2.76, 95% CI: -4.95 to -0.56, p = 0.0137), and left ventricular filling pressure (MD: -0.59, 95% CI: -1.07 to -0.10, p = 0.0189). However, empagliflozin treatment did not yield a statistically significant change in left ventricular ejection fraction (MD: 1.21, 95% CI: -0.05 to 2.48, p = 0.0603) nor a significant change in left ventricular end-diastolic volume (MD: -4.49, 95% CI: -14.24 to 5.26, p = 0.37), left ventricular end-systolic volume (MD: -5.19, 95% CI: -10.77 to 0.39, p = 0.0682), and left ventricular end-diastolic volume index (MD: -2.20, 95% CI: -4.59 to 0.19, p = 0.0718). Empagliflozin provides favorable effects on left cardiac structural parameters in ACS patients. This suggests a potential role for SGLT2 inhibitors as adjunctive therapy in ACS management, warranting further investigation into their mechanisms and long-term clinical outcomes.

## Introduction and background

Acute coronary syndrome (ACS) is a complex spectrum of acute ischemic heart disorders that includes unstable angina, non-ST-segment elevation myocardial infarction (NSTEMI), and ST-segment elevation myocardial infarction (STEMI) [1]. Despite significant advancements in the identification and management of acute coronary syndromes, the primary cause of mortality worldwide is still cardiovascular diseases, with ischemic heart disease constituting around half of these fatalities [2]. The broad consequences of ACS go beyond the person, putting pressure on economies, social well-being, and healthcare systems [3,4].

Recently, the class of drugs known as sodium-glucose cotransporter 2 (SGLT2) inhibitors has drawn interest during the search for efficacious management of ACS, in addition to being used for type 2 diabetic mellitus (T2DM) [5]. SGLT2 inhibitors specifically block SGLT2 receptors in the proximal renal tubules to decrease renal glucose reabsorption and raise glucose excretion in the urine [6]. By employing immunocytochemistry and western blotting methods, it was recently shown that smooth muscle cells (SMCs) and endothelial cells (ECs) expressed SGLT2 and were inhibited with empagliflozin, in particular [7,8]. The finding might suggest a connection between SGLT2 and the onset and progression of coronary heart disease (CHD) and restenosis, aside from its primary glycemic control mechanism.

Patients with CHD have been shown to benefit from empagliflozin in multiple clinical trials [9,10]. All these promising roles of SGLT2 inhibitors in cardiovascular disease management were reflected in the latest European Society of Cardiology (ESC) guidelines, which state that SGLT2 inhibitors should be the basis of pharmacological management for heart failure, independent of a patient's diabetic status [11]. According to a recent meta-analysis, individuals on SGLT2 inhibitors had a significantly lower risk of major adverse cardiovascular events (MACEs), all-cause mortality, and cardiovascular mortality when compared to the control group [5]. To go deeper into the role of SGLT2 inhibitors in treating ACS patients, we looked into the implications of empagliflozin on left cardiac structural parameters among ACS patients in both diabetic and non-diabetic populations by conducting a systematic review and meta-analysis.

## Review

Methods

Protocol Registration

This systematic review and meta-analysis adhered to the Preferred Reporting Items for Systematic Reviews and Meta-Analyses (PRISMA) 2020 guidelines [12,13]. This study's protocol was registered prospectively in Open Science Framework (https://osf.io/vd978) [14].

Data Sources and Search Strategy

A thorough search was carried out on PubMed, Scopus, Cochrane Library, and Web of Science (last update, May 2024). In addition, ClinicalTrials.gov was consulted to identify any potentially overlooked trials. No filters were applied, and all studies involving human subjects, regardless of publication language, were selected. The full search strategy showing the terms used is (“Empagliflozin” OR “Jardiance” OR “BI-10773” OR “BI 10773 “ OR “BI10773” OR “SGLT2-I” OR “SGLT 2 I” OR “SGLT 2 inhibitors” OR “Sodium Glucose Transporter 2 inhibitors” OR “Sodium Glucose Transporter”) AND (“Coronary Syndrome” OR “Myocardial Infarction” OR “Heart Attack” OR “Cardiovascular Stroke” OR “Acute Coronary Syndrome” OR “MI” OR “ACS” OR “Myocardial Ischemia” OR “Ischemic Heart Disease” OR “ST Elevation Myocardial Infarction” OR “ST Segment Elevation Myocardial Infarction” OR “ST Elevated Myocardial Infarction” OR “STEMI” OR “Non ST Elevation Myocardial Infarction” OR “Non-ST-Elevation Myocardial Infarction” OR “Non ST Elevated Myocardial Infarction” OR “Non-ST Elevated Myocardial Infarction” OR “NSTEMI” OR “Unstable Angina” OR “unstable angina pectoris” OR “Preinfarction Angina” OR “Angina at Rest” OR “Myocardial Preinfarction Syndrome”). Furthermore, reference screening of the included trials was done to find any missed RCT.

Eligibility Criteria and Study Selection Process

The retrieved articles were managed, and duplicates were removed using EndNote 21. The remaining papers' title and abstract screening was conducted by two independent researchers using Rayyan online software to select the most relevant studies [15]. The full texts were then examined to assess their applicability.

We focused on individuals diagnosed with acute coronary syndrome (ACS) diagnosis and included participants aged 18 years and above. We limited our scope to trials published in English. Furthermore, we included patients diagnosed with ACS, regardless of their diabetic or non-diabetic status. Our intervention of interest was empagliflozin, and we compared its efficacy against a placebo. The primary outcome we assessed was hemodynamic left cardiac parameters, and we exclusively included randomized controlled trials (RCTs).

Data Extraction

The process of extracting data was carried out by two authors working independently to ensure a thorough and unbiased assessment. The data extraction sheet was thoughtfully structured into three distinct sections to capture the essential information from each study. The first section encompassed a summary of each study, including study ID, title, sample size, last name of the first author, publishing year, inclusion and exclusion standards, group numbers, outcomes, conclusion, and treatment specifics. The second section captured the studies' basic characteristics, mainly age, gender, and structural parameters. Finally, the third section detailed the studies' outcomes.

Quality Assessment

The Cochrane risk-of-bias tool for randomized trials (RoB 2), version 2, was used to assess the methodological quality of randomized controlled trials [16]. Each study's quality was evaluated by two different authors separately, and disagreements were addressed and worked out by consensus. Because there were less than 10 included studies, we were unable to evaluate the publication bias [17].

Statistical Analysis

R (Version 4.3) was utilized for the statistical analysis [18]. The meta-analyses were performed using the "meta" package [19]. Using the fixed or random effects model, we only had continuous outcomes, compared using mean difference (MD) with a 95% confidence interval (CI). To evaluate heterogeneity, we used the Chi-square, an alpha threshold of less than 0.1, to indicate considerable heterogeneity. To address the heterogeneity, we used leave-one-out sensitivity analysis.

Results

Literature Search

**Figure 1:**
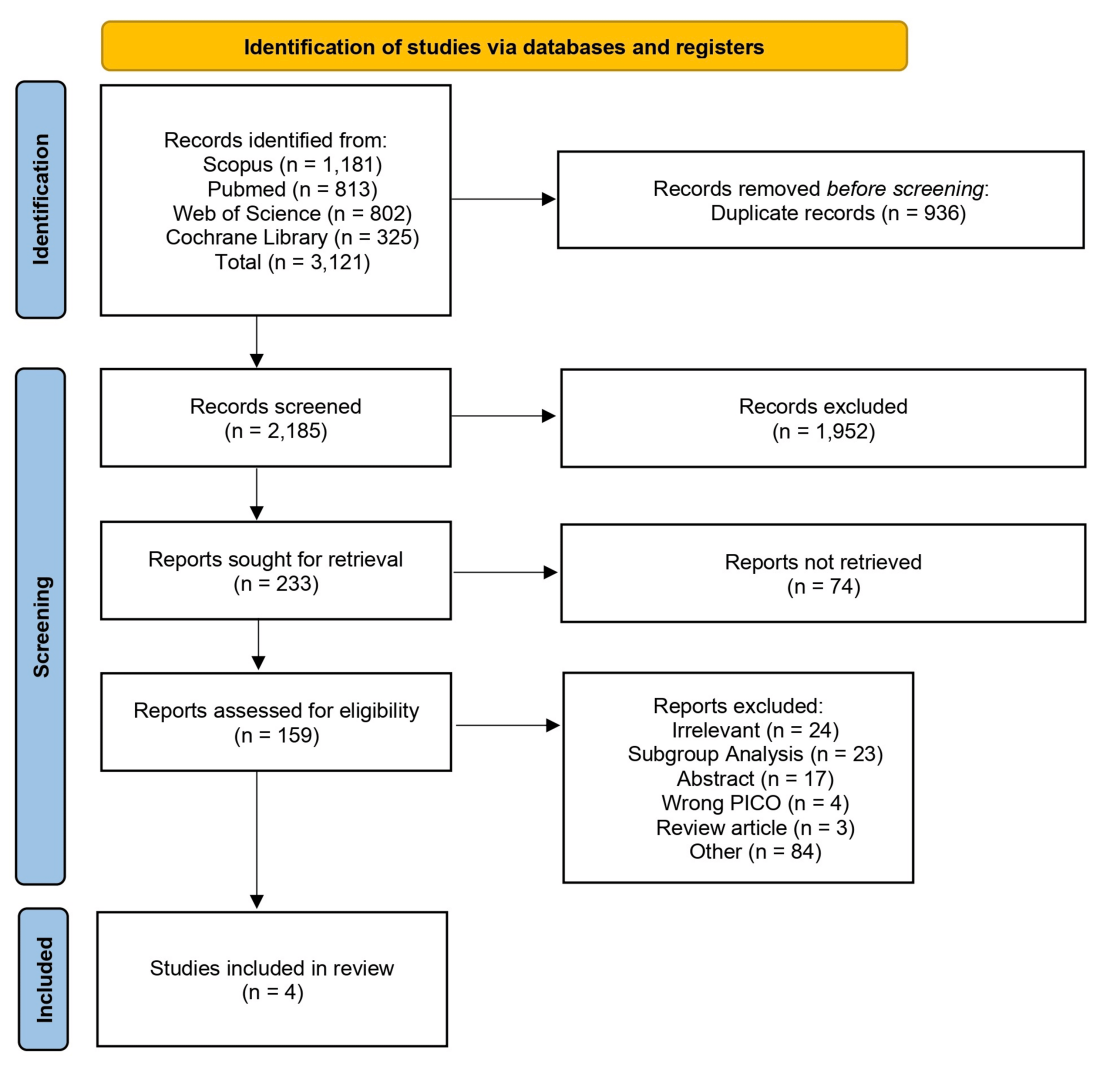
PRISMA flowchart. PRISMA:  Preferred Reporting Items for Systematic Reviews and Meta-Analyses.

Across all databases, 3,121 studies were obtained. After removing duplicates and title and abstract screening, 159 were eligible for full-text screening. A total of four studies were best matched our eligibility criteria and included for qualitative and quantitative analysis. We found no missed studies when manually searching the references of the included studies. A detailed flowchart for the selection of clinical trials is displayed in Figure 1.

Characteristics of the Included Studies

The meta-analysis incorporated four RCTs [20-23], which comprised 701 patients with ACS, 346 of whom were in the group that received empagliflozin, and 355 of them received a placebo. The participants' mean age was 61, ranging from 54 to 68 years. The trials' average follow-up period was six months, except Lundin et al. [20], which had a follow-up duration of 10 months. The referenced studies were carried out across multiple countries, namely Iran, Canada, Sweden, and several others. A detailed summary of the studies and baseline characteristics of the participants can be found in Tables 1, 2, respectively.

**Table 1:** Summary of included studies. RCT: randomized controlled trial; DM: diabetes mellitus; STEMI: ST-segment elevation myocardial infarction; NSTEMI: non-ST-segment elevation myocardial infarction; MACE: major adverse cardiovascular event; ACS: acute coronary syndrome; T2DM: type 2 diabetes mellitus; LVMi: left ventricular mass index; MI: myocardial infarction; PCI: percutaneous coronary intervention; eGFR: estimated glomerular filtration rate; NT-proBNP: N-terminal pro-B-type natriuretic peptide; HbA1c: hemoglobin A1c; IGT: impaired glucose tolerance; CMR: cardiac magnetic resonance; CABG: coronary artery bypass grafting.

Study	Year	Design	Country	No. of Centers	Empagliflozin Dose	Total Participants Number	Follow-up Duration	Inclusion Criteria	Primary Outcomes	Secondary Outcomes	Conclusion
Adel et al. [23]	2022	RCT	Iran	2	10 mg	106 (randomized), 93 (included in analysis)	6 months	1) Over 18 years. 2) Previous diagnosis of DM and acute coronary syndrome (STEMI, NSTEMI, unstable angina).	Major cardiovascular complications (MACE) include death from cardiovascular causes, hospitalization for heart failure, recurrent angina, stroke, non-fatal MI, transient ischemic attack, and all-cause mortality.	-	Adding empagliflozin to the standard care of individuals with ACS who are diabetic after PCI did not significantly reduce negative cardiovascular outcomes during the 6-month follow-up period.
von Lewinski et al. [22]	2022	RCT	Austria	11	10 mg	476 (randomized), 476 (included in analysis)	26 weeks	1) 18 to 80 years of age. 2) Verified acute major MI (creatine kinase >800 IU/L); high-sensitivity troponin T (or troponin I) level >10 times normal upper limit. 3) eGFR > 45 mL/min/1.73 m².	NT-proBNP level change over 26 weeks.	1) Variations in NT-proBNP levels between week 6 and randomization. 2) Variations in body weight, ketone body, and HbA1c, and echocardiographic parameters for diastolic dysfunction, left ventricular end-systolic volume (LVESV), and end-diastolic volume (LVEDV) from randomization to weeks 6 and 26. 3) Hospitalizations for heart failure or other reasons, length of hospital stay, and overall mortality were additional exploratory goals.	Empagliflozin was linked to a much higher reduction in NT-proBNP over a 26-week period in individuals who had recently experienced a myocardial infarction. A notable improvement in echocardiographic functional and structural characteristics supported this improvement.
Lundin et al. [20]	2022	RCT	Sweden	1	25 mg	42 (randomized), 42 (included in analysis)	10 months	1) Over 18 years. 2) Had recently been diagnosed with IGT or T2DM. 3) Had unstable angina or acute MI for the prior six months.	LVEDV change from baseline to seven months.	1) Systolic and diastolic LV function. 2) Coronary flow reserve. 3) Extracellular volume in non-infarcted myocardium. 4) arterial stiffness changes.	The CMR and echocardiographic parameters that represent LV function were unaffected by empagliflozin.
Verma et al. [21]	2019	RCT	Canada	1	10 mg	97 (randomized), 90 (included in analysis)	6 months	1) Age 40-80 years. 2) HbA1c 6.5% to 10%. 3) Known coronary disease (myocardial infarction history or prior coronary revascularization using CABG or PCI. 4) GFR ≥ 60 mL/min/1.73 m². 5) Had been stable on antihypertensive drugs 2 months before enrolment.	The change of LVMi in 6 months.	1) LVEDV or LVEDVi. 2) LVESV or LVESVi. 3) LVEF. 4) NT-proBNP.	After 6 months, empagliflozin was linked to a significant decrease in LVMi in individuals with T2DM and coronary artery disease.

**Table 2:** Baseline characteristics. Unless otherwise indicated, values are n (%) or mean ± SD. LVMi: left ventricular mass index; LVESVi: left ventricular end-systolic volume index; LVEDVi: left ventricular end-diastolic volume index; LVESV: left ventricular end-systolic volume; LVEDV: left ventricular end-diastolic volume; LVEF: left ventricular ejection fraction; E/eʹ: left ventricular filling pressure.

Study	Arms	Age (Years)	Male	Type 2 Diabetes	Non-Diabetic	LVMi (gm/m2)	LVESVi (ml/m2)	LVEDVi (ml/m2)	LVESV (ml)	LVEDV (ml)	EF (%)	E/eʹ
Adel et al. [23]	Empagliflozin	54.8 ± 14.2	27 (60%)	45 (100%)	-	-	-	-	-	-	41.67 ± 15.32	-
	Placebo	57.9 ± 12.8	29 (60.4%)	48 (100%)	-	-	-	-	-	-	43.75 ± 10.5	-
von Lewinski et al. [22]	Empagliflozin	57.67 ± 8.95	195 (82.28%)	30 (13%)	207 (87%)	-	30.67 ± 8.95	58 ± 14.92	61.67 ± 20.88	117 ± 34.31	-	8.67 ± 2.98
	Placebo	58 ± 9.7	197 (82.43%)	33 (14%)	206 (86%)	-	29.33 ± 9.7	56.33 ± 12.68	59.67 ± 20.14	113.33 ± 31.325	48.67 ± 8.2	9.33 ± 1.49
Lundin et al. [20]	Empagliflozin	67 ± 8	16 (80%)	-	-	42.7 ± 5.2	73.05 ± 16.67	32.06 ± 13.46	-	146 ± 33	-	13 ± 4
	Placebo	68 ± 8	18 (81.82%)	-	-	41.69 ± 2.69	70.05 ± 16.84	26.92 ± 12	-	141 ± 39	-	11 ± 3
Verma et al. [21]	Empagliflozin	63.33 ± 9.17	44 (90%)	49 (100%)	-	57.6 ± 9.9	27.1 ± 10.5	63.3 ± 15.5	53 ± 20.8	124.1 ± 33	58 ± 7.5	-
	Placebo	64 ± 12.23	46 (96%)	48 (100%)	-	61.3 ± 12.2	32.3 ± 11.8	71.4 ± 15.4	62.5 ± 26	138.4 ± 39.1	55.5 ± 8.7	-

Risk of Bias Assessment

**Figure 2:**
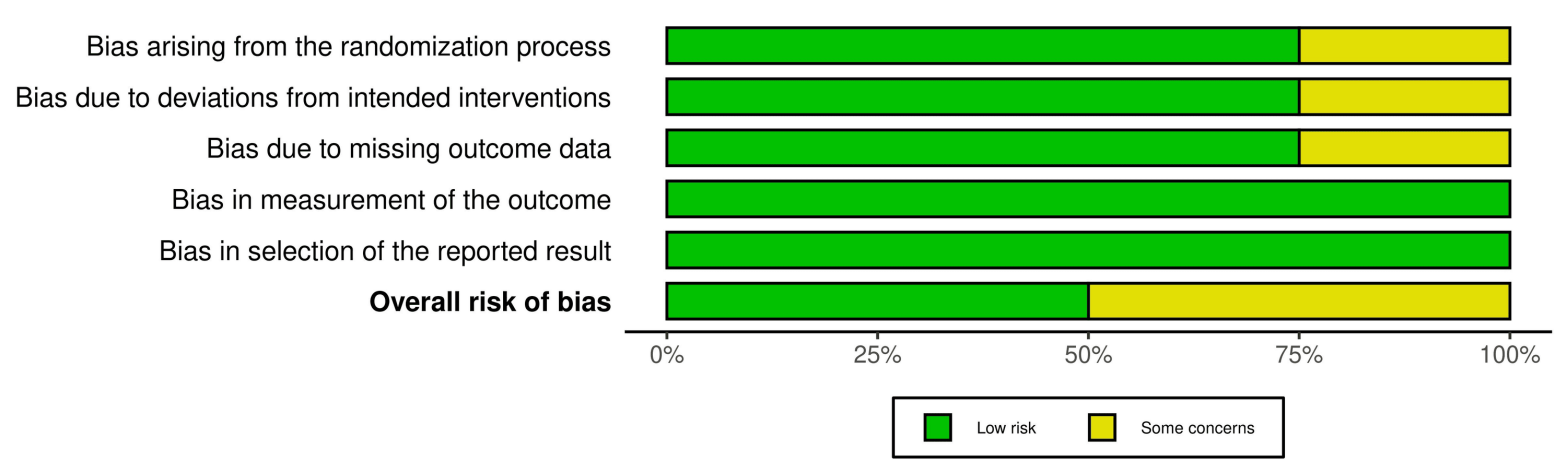
Risk of bias assessment.

We assessed the four RCTs with the Cochrane Risk of Bias 2 tool and determined that two trials had a low risk of bias, and the other two had a moderate risk of bias, as presented in Table 3 and Figure 2.

**Table 3:** Risk of bias assessment of the included studies.

Study	Bias Arising From the Randomization Process	Bias Due to Deviations From Intended Intervention	Bias Due to Missing Outcome Data	Bias in Measurement of the Outcome	Bias in Selection of the Reported Result	Overall Risk of Bias
Adel et al. [23]	Low risk	Some concerns	Some concerns	Low risk	Low risk	Some concerns
von Lewinski et al. [22]	Low risk	Low risk	Low risk	Low risk	Low risk	Low risk
Lundin et al. [20]	Some concerns	Low risk	Low risk	Low risk	Low risk	Some concerns
Verma et al. [21]	Low risk	Low risk	Low risk	Low risk	Low risk	Low risk

Cardiac Structural Parameters Results

**Figure 3:**
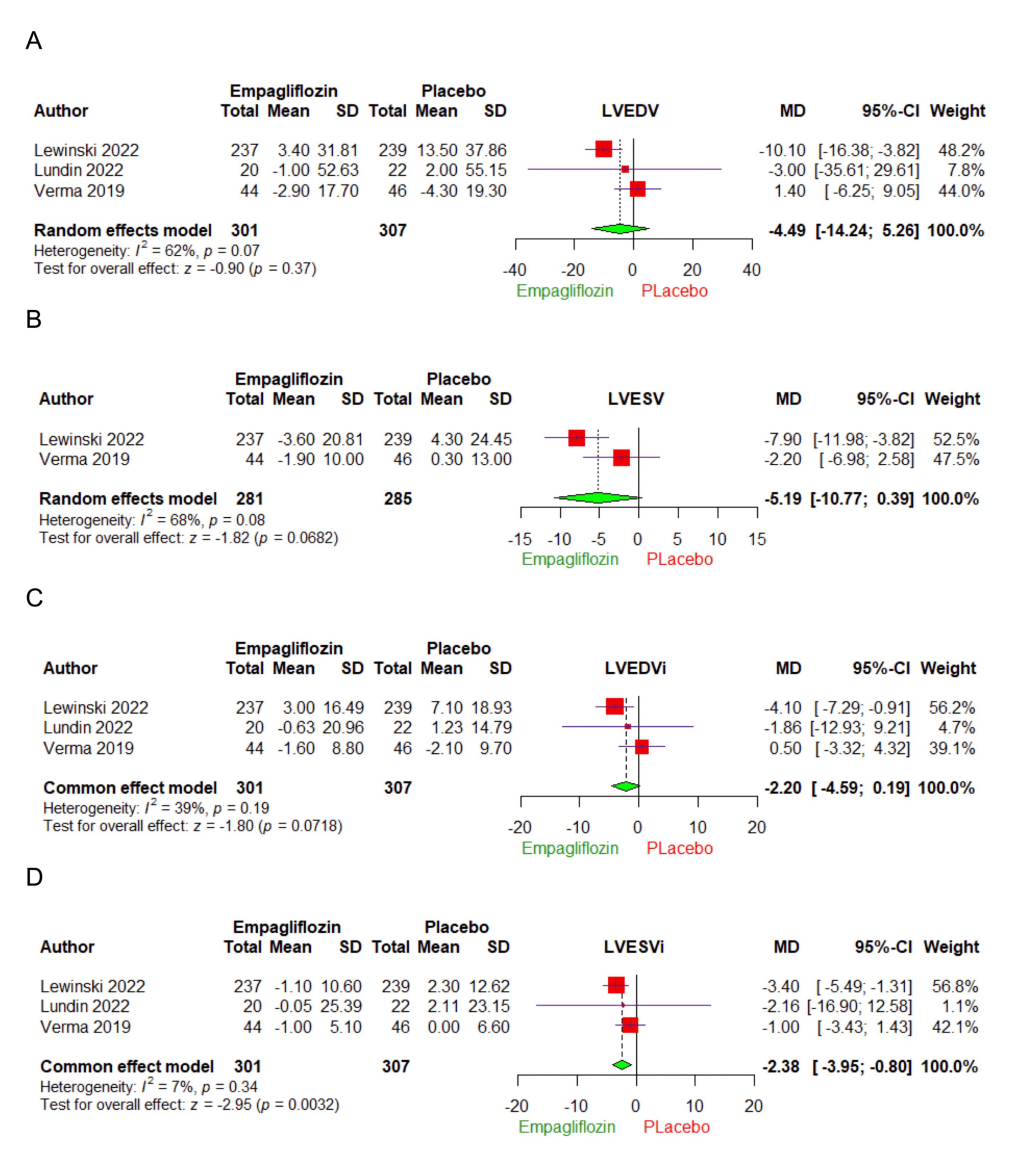
Forest plots demonstrating the effect of Empagliflozin on (A) left ventricular end-diastolic volume (LVEDV), (B) left ventricular end-systolic volume (LVESV), (C) left ventricular end-diastolic volume index (LVEDVi), and (D) left ventricular end-systolic volume index (LVESVi). SD: standard deviation, CI: confidence interval, MD: mean difference.

**Figure 4:**
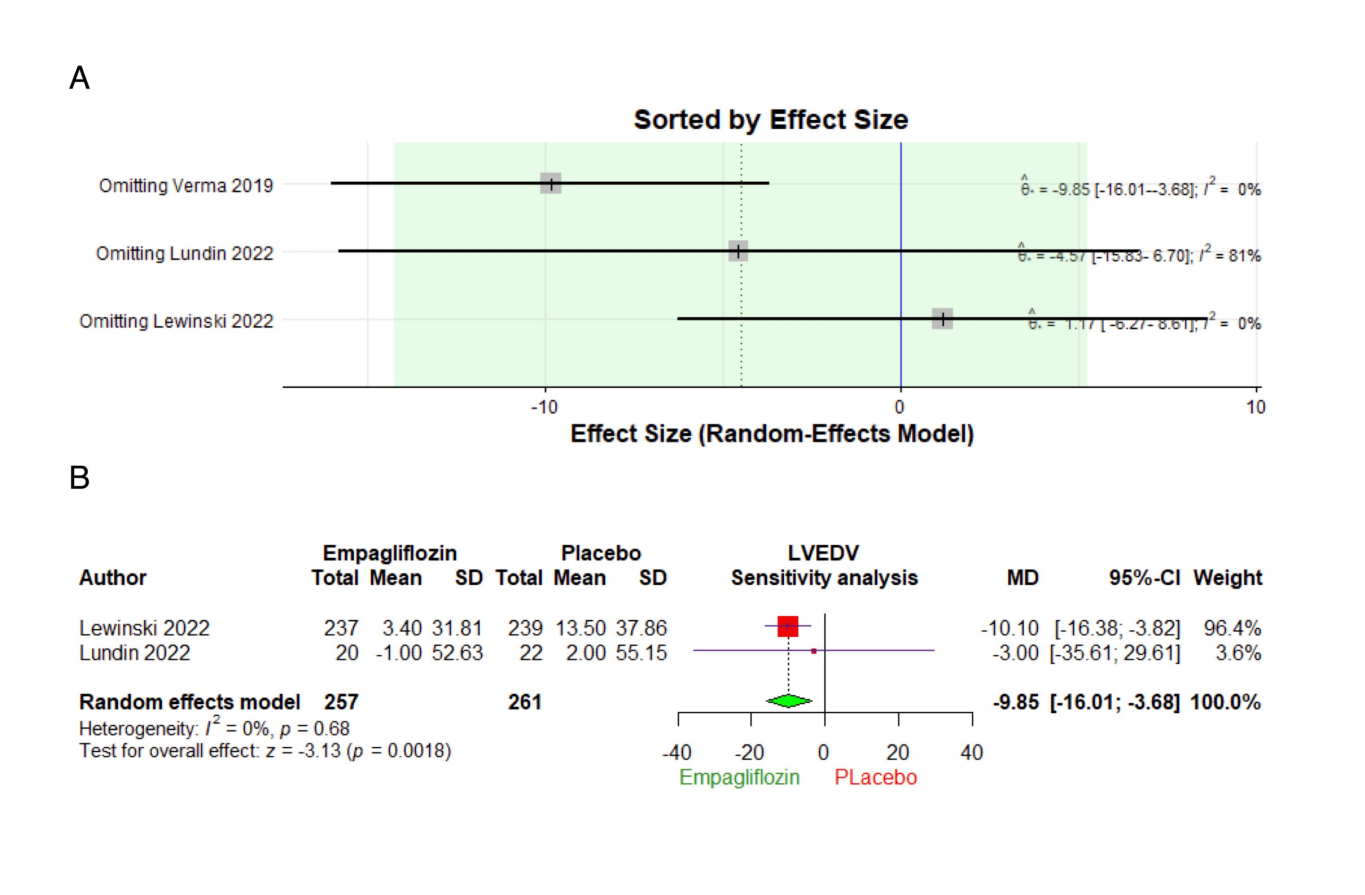
Leave-one-out analysis and sensitivity analysis of left ventricular end-diastolic volume (LVEDV). (A) Leave-one-out analysis for LVEDV, (B) Forest plot showing the sensitivity analysis of LVEDV by excluding Verma et al. [21] 2019. SD: standard deviation, CI: confidence interval, MD: mean difference.

Left ventricular end-diastolic volume (LVEDV): Three studies comprising 608 patients were involved in the analysis of LVEDV (Figure 3A). Compared with the placebo group, Empagliflozin treatment did not cause a statistically significant reduction in LVEDV (MD: -4.49, 95% CI: -14.24 to 5.26, p = 0.37). The combined studies were heterogeneous (I² = 62%, p = 0.07). The heterogeneity was properly addressed by excluding Verma et al. [21] from the analysis (I² = 0%, p = 0.68) (Figures 4A, 4B).

Left ventricular end-systolic volume (LVESV): Two studies comprising 566 patients were involved in the analysis of LVESV (Figure 3B). Compared with the placebo group, Empagliflozin treatment did not cause a statistically significant reduction in LVESV (MD: -5.19, 95% CI: -10.77 to 0.39, p = 0.0682). The combined studies were heterogeneous (I² = 68%, p = 0.08).

Left ventricular end-diastolic volume index (LVEDVi): Three studies comprising 608 patients were involved in the analysis of LVEDVi (Figure 3C). Compared with the placebo group, Empagliflozin treatment did not cause a statistically significant reduction in LVEDVi (MD: -2.20, 95% CI: -4.59 to 0.19, p = 0.0718). The combined studies were homogenous (I² = 39%, p = 0.19).

Left ventricular end-systolic volume index (LVESVi): Three studies comprising 608 patients were involved in the analysis of LVESVi (Figure 3D). Compared with the placebo group, Empagliflozin treatment caused a statistically significant reduction in LVESVi (MD: -2.38, 95% CI: -3.9 to -0.80, p = 0.0032). The combined studies were homogenous (I² = 7%, p = 0.34).

**Figure 5:**
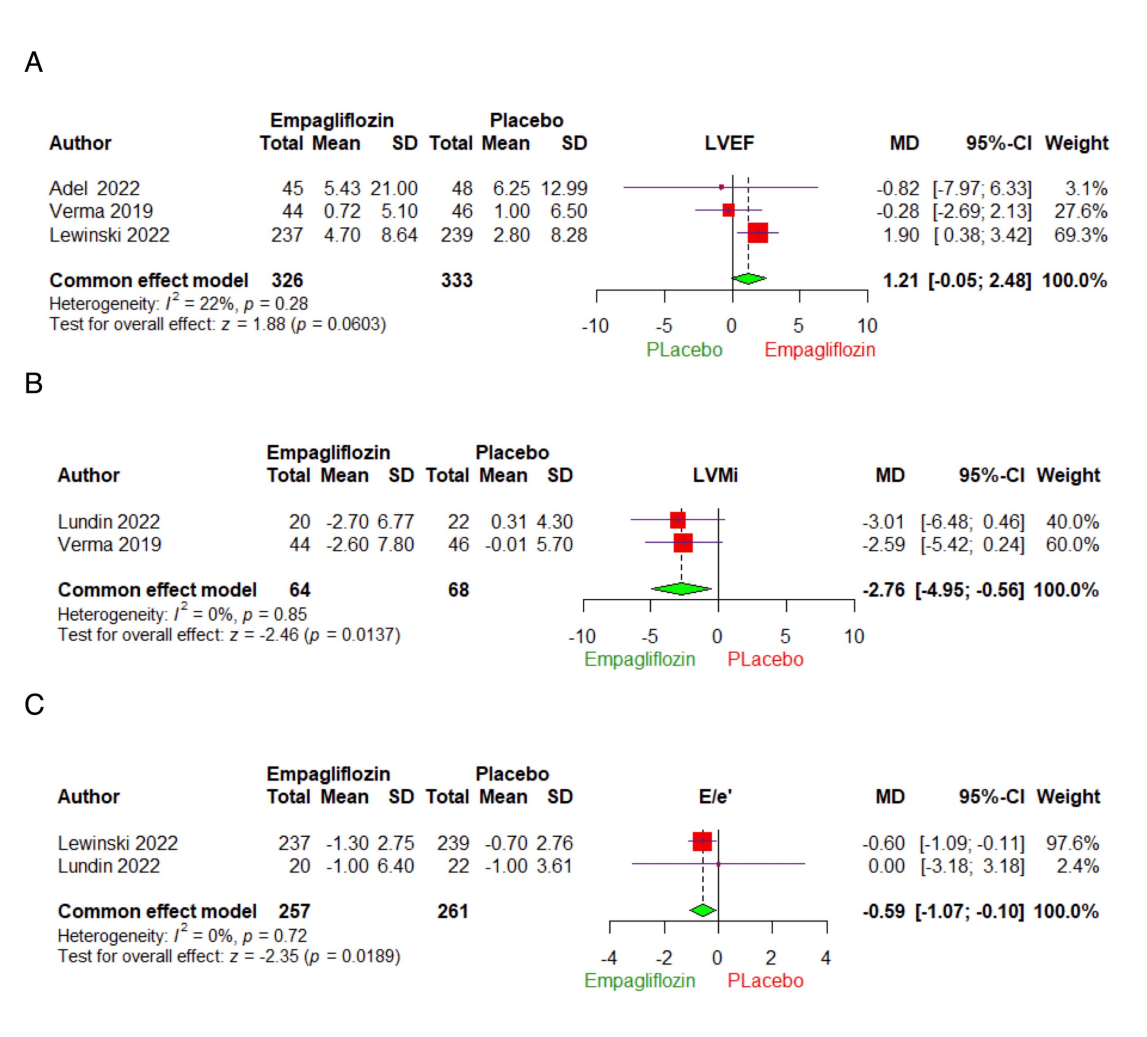
Forest plots demonstrating the effect of Empagliflozin on (A) left ventricular ejection fraction (LVEF), (B) left ventricular mass index (LVMi), and (C) left ventricular filling pressure (E/e'). SD: standard deviation, CI: confidence interval, MD: mean difference.

Left ventricular ejection fraction (LVEF): Three studies comprising 659 patients were involved in the analysis of LVEF (Figure 5A). Compared with the placebo group, Empagliflozin treatment did not cause a statistically significant increase in LVEF (MD: 1.21, 95% CI: -0.05 to 2.48, p = 0.0603). The combined studies were homogenous (I² = 22%, p = 0.28).

Left ventricular mass index (LVMi): Two studies comprising 132 patients were involved in the analysis of LVMi (Figure 5B). Compared with the placebo group, Empagliflozin treatment caused a statistically significant reduction in LVMi (MD: -2.76, 95% CI: -4.95 to -0.56, p = 0.0137). The combined studies were homogenous (I² = 0%, p = 0.85).

Left ventricular filling pressure (E/e'): Two studies comprising 518 patients were involved in the analysis of E/e' (Figure 5C). Compared with the placebo group, Empagliflozin treatment caused a statistically significant reduction in E/e' (MD: -0.59, 95% CI: -1.07 to -0.10, p = 0.02). The combined studies were homogenous (I² = 0%, p = 0.72).

Discussion

Key Findings

This meta-analysis is the initial thorough examination of empagliflozin's impact on diverse cardiac parameters in individuals with acute coronary syndrome, regardless of their diabetic status. The study assessed several primary outcomes, including LVEDD, LVESD, LVEDVi, LVESVi, LVEF, LVMi, and E/e'. In the empagliflozin-treated group, no significant improvements were observed in the assessed cardiac parameters compared to the control group, except for LVESVi, LVMi, and E/e', which demonstrated a substantial variance between the two groups.

Explanation of Our Findings

The cardiovascular benefits of SGLT2 inhibitors, particularly empagliflozin, are evident in patients with and without diabetes [24], regardless of whether their LVEF is reduced or preserved [25,26]. Several trials and meta-analyses have shown the superiority of empagliflozin over other SGLT2 inhibitors in cardiovascular outcomes improvement [27]. Several factors contribute to the beneficial effects on cardiovascular outcomes. The decline in occurrences associated with heart failure is one important aspect, and it becomes apparent shortly after treatment initiation. In addition, enhanced sodium excretion, increased osmotic diuresis, improved myocardial energetics, and less interstitial edema are elements that could lead to cardiovascular benefits [28].

Regarding LVMi, our study showed a favorable effect of empagliflozin in reducing LVMi. This decrease indicates a potential protective role of empagliflozin against left ventricular myocardial infarction. This finding aligns with the Verma et al. study [21], which indicates that individuals with both T2DM and coronary artery disease, treated with empagliflozin in addition to standard antihyperglycemic medication, had a notable reduction in LVMi as assessed through cardiac magnetic resonance imaging (CMRI). In this study, during the six-month duration, individuals in the empagliflozin cohort demonstrated an average decrease of 3.35 g/m^2^ compared to those in the placebo arm. The findings from this trial mark the first randomized clinical evidence in humans illustrating the ability of the SGLT2 inhibitor empagliflozin to induce regression of LV mass. This is noteworthy given that regression of LVMi is a pivotal factor in determining cardiovascular disorders and cardiovascular-related deaths. Importantly, the study observed beneficial LVMi early, within a treatment duration of six months. This is in line with the EMPA-REG OUTCOME trial [29], which showed the early divergence observed in the Kaplan-Meier curves for heart failure hospitalization and cardiovascular mortality. Notably, in Verma et al. [21], it was noted that individuals exhibiting the highest baseline LVMi experienced the most significant regression in left ventricular mass [21]. Particularly, among participants with an initial LVMi exceeding 60 g/m^2^, a 7.3 g/m^2^ reduction in LVMi was documented. The specific processes responsible for the decrease in the thickness of the wall following empagliflozin treatment are not yet fully understood. This reduction could be due to a decrease in cardiomyocyte mass, alterations in interstitial aqueous content, or a combination of these factors. Despite the blood pressure-lowering properties of empagliflozin, further analysis indicates that the decline in LVMi is not primarily driven by changes in blood pressure. While a positive correlation was found between baseline systolic blood pressure and LVMi, there was no significant association between changes in continuous blood pressure monitoring over 24 hours and changes in LVM over the six-month monitoring period. These findings indicate that other mechanisms apart from blood pressure lowering could play a significant role in the regression of LVMi following empagliflozin treatment.

Our study demonstrated that empagliflozin led to a reduction in LVESVi compared to placebo, suggesting a favorable effect on ventricular remodeling and potentially leading to improved cardiac function. This result aligns with the findings of von Lewinski et al. [22]. Furthermore, the improved E/e' ratio in the empagliflozin group indicates a reduction in left ventricular filling pressures, which is a positive outcome associated with improved diastolic function. This finding is also consistent with the results of von Lewinski et al. [22].

Regarding LVEDVi, there was no discernible difference between the placebo and empagliflozin groups in our study, suggesting that empagliflozin may not significantly influence this specific cardiac parameter in patients with coronary syndrome. This finding aligns with the outcomes reported by Verma et al. and von Lewinski et al. [21,22].

Regarding LVEDV, our results showed no significant difference between the two groups, consistent with the findings of Lundin et al. [20] in their randomized, double-blind, placebo-controlled, investigator-initiated trial, 42 patients with recent myocardial infarction or unstable angina, and newly detected impaired glucose tolerance or type 2 diabetes, were randomized to receive 25 mg of empagliflozin daily (n = 20) or placebo (n = 22) in addition to ongoing therapy. They underwent oral glucose tolerance tests, stress-perfusion CMRI, and echocardiography at three different times: before randomization, after seven months on the drug, and three months after stopping the drug.

The use of both CMRI and echocardiography, which revealed similar outcomes, strengthens these results. The extended follow-up period and the repeated investigations after stopping the drug further demonstrate the stability of these observations. The lack of significant difference in our study regarding LVEDV might be attributed to the fact that LV mass, which did show a significant difference in our results, could be a more accurate endpoint in patients with reduced LV ejection fraction compared to LV end-diastolic volume [20]. Our findings did not align with those of von Lewinski et al. [22], who carried out a double-blind, multicenter trial with 476 patients experiencing acute myocardial infarction. In their research, after undergoing percutaneous coronary intervention, participants were randomly assigned to receive 10 mg of empagliflozin or a placebo, administered daily for 72 hours. However, when performing leave-one-out analysis and omitting Verma et al. [21], a significant statistical change was observed.

In terms of LVESV, our findings showed no significant difference between the two groups, which is in agreement with Verma et al. [21]; by using CMRI in their study, no changes were found in ventricular volumes with empagliflozin treatment, a technique proven to detect end-systolic and end-diastolic volume changes as minimal as 5 and 10 mL, respectively. Hence, the lack of observed change, not only in LVESV but also in LVEDV, cannot be attributed to measurement insensitivity. Since only two trials reported the outcome LVESV, we could not resolve the heterogeneity.

Moreover, the trial of Lundin et al. [20] revealed that SGLT2 inhibition benefits patients with heart failure or those at risk of heart failure, irrespective of LV ejection fraction and the presence of T2DM. Therefore, this treatment does not seem to provide consistent benefits for individuals with normal cardiac dimensions and function after acute coronary syndrome. Additionally, the cardiac effects of SGLT2 inhibition may be less effective in patients with newly diagnosed dysglycemia, as observed in their study population, which primarily included individuals with IGT rather than T2DM.

Our results demonstrated no significant difference in LVEF between the two groups, aligning with the findings of Lundin et al. [20], which indicated that empagliflozin did not affect indexed LV volumes or LV ejection fraction. Similarly, Adel et al. [23] conducted a double-blind controlled clinical trial involving 93 diabetic patients with ACS who underwent percutaneous coronary intervention (PCI). In this trial, patients were randomly assigned to receive either empagliflozin (10 mg once daily) or placebo for six months post-PCI, in addition to standard hypoglycemic treatments.

Consistency and Inconsistency With Previous Findings

Currently, no meta-analysis exists specifically examining the comparative efficacy of empagliflozin against placebo following acute coronary syndrome. Hence, this meta-analysis serves to provide knowledge about this gap in the literature.

Robustness and Weaknesses Points

This study represents the first comprehensive analysis focusing on the impact of empagliflozin on cardiac parameters in patients with acute coronary syndrome. Despite its contributions, the study has some limitations, including limited large-scale randomized trials and a primary focus on patients with coronary syndrome, which would be an obstacle to the findings' applicability to broader populations. Additionally, heterogeneity was observed in some outcomes. To address this heterogeneity, a sensitivity analysis was conducted on outcomes reported in three trials, revealing resolution when excluding the study by Verma et al. [21]; this heterogeneity may be attributed to changes in medication among participants during the study, with a notable percentage involving medications not listed in the baseline characteristics. The potential impact of these medication changes on outcomes in this cohort warrants further investigation.

Practical Applications and Future Investigations

The results offer important insights into how empagliflozin could enhance left ventricular parameters in this at-risk group in clinical settings. Multicenter studies measuring the alterations in cardiac parameters after the use of empagliflozin, in particular, and SGLT2 inhibitors, in general, are needed. We recommend longer follow-up durations and larger sample sizes for future studies.

## Conclusions

This meta-analysis offers a thorough assessment of empagliflozin's effects on various cardiac parameters in patients with acute coronary syndrome, with or without diabetes. The results suggest that empagliflozin treatment improved several important cardiac parameters, including LVESVi, LVMi, and E/e' ratio, which are influential in assessing cardiac structural performance. These improvements highlight empagliflozin's potential role in managing patients with ACS, offering benefits beyond its glucose-lowering effects.
